# Association of serum 25-hydroxyvitamin D with urinary incontinence in elderly men: evidence based on NHANES 2007-2014

**DOI:** 10.3389/fendo.2023.1215666

**Published:** 2023-09-08

**Authors:** Li Liu, Mingming Xu, Hang Zhou, Xuexue Hao, Xiangyu Chen, Xiaoqiang Liu

**Affiliations:** Department of Urology, Tianjin Medical University General Hospital, Tianjin, China

**Keywords:** urinary incontinence, 25-hydroxyvitamin D, NHANES, elderly men, vitamin D deficiency

## Abstract

**Background:**

The correlation between serum 25-hydroxyvitamin D (25(OH)D) and different sub-types of urinary incontinence in elderly men continues to be uncertain. Hence, we performed this research to evaluate whether serum 25(OH)D levels are correlated with urinary incontinence among elderly men.

**Methods:**

The present study incorporated the male population aged 50 years and above from four cycles of the NHANES database spanning from 2007 to 2014, for the purpose of analysis. The assessment of urinary incontinence was carried out through a correlation questionnaire, while standardized liquid chromatography-tandem mass spectrometry (LC-MS/MS) was adopted to quantify serum 25(OH)D. A weighted multi-factorial logistic regression analysis was carried out to ascertain and investigate any potential correlation that may exist between serum 25(OH)D and urinary incontinence in senior males.

**Results:**

Ultimately, a sum of 4663 elderly men were involved in our analysis. The outcomes of the univariable analysis illustrated that the group with vitamin D deficiency exhibited augmented odds of all three urinary incontinence types in comparison to the vitamin D-sufficient group. After accounting for age, race, and BMI, no appreciable variations in the outcomes were noticed. However, after accounting for all covariates, only SUI (OR = 1.677; 95% confidence interval (CI) = 1.074–2.618) and MUI (OR = 1.815; 95% confidence interval (CI) = 1.010–3.260) demonstrated statistical significance.

**Conclusion:**

Decreased serum 25(OH)D levels were connected with stress urinary incontinence and mixed urinary incontinence in elderly men.

## Introduction

Urinary incontinence (UI) is a prevalent disorder characterized by the uncontrollable leakage of urine (1). This disorder has a profound effect on the affected individual’s standard of living and imposes a huge cost on society (2). Stress urinary incontinence (SUI), urge urinary incontinence (UUI), and mixed urinary incontinence (MUI) are the three most common forms of urinary incontinence (3). While UI is more prevalent in women, it is not limited to this gender and is also widespread in a significant percentage of men, particularly in older age groups. According to the European Association of Urology guidelines, UI affects approximately 11% of men between the ages of 60–64 years and up to 31% of men aged 85 years (1). Hence, the impact of UI on men, especially the elderly, should not be overlooked.

Vitamin D is a fat-soluble vitamin synthesized primarily by human skin after exposure to ultraviolet radiation and secondarily by food or other forms of supplementation (4). Its primary function is to maintain calcium and phosphorus metabolism and bone health in the body, with its main form in the body being 25-hydroxyvitamin D (25(OH)D) (5). A serum level of 25(OH)D below 50 nmol/L is typically considered a sign of vitamin D deficiency (6, 7). Vitamin D deficiency is a pervasive condition affecting a significant population worldwide. Studies have shown that approximately 1 billion people are impacted by vitamin D insufficiency or deficiency, particularly among the elderly population (8). Notably, there is a clear correlation between vitamin D deficiency and bone diseases (9). Furthermore, research has revealed that vitamin D insufficiency or deficiency is also linked to various conditions such as muscle weakness, tumors, depression, metabolic diseases, and cardiovascular diseases (8, 10–12).

Numerous studies have demonstrated a significant correlation between vitamin D deficiency and urinary incontinence in women (13, 14). Interestingly, a study has even suggested that vitamin D supplementation may improve urinary incontinence in premenopausal women (15). However, relatively little is known regarding the association between vitamin D deficiency and urinary incontinence in older men. Accordingly, the primary aim of this research endeavor is to explore the correlation between vitamin D deficiency and urinary incontinence in older men, using data obtained from the National Health and Nutrition Examination Survey (NHANES).

## Methods

### Study participants

**Figure 1:**
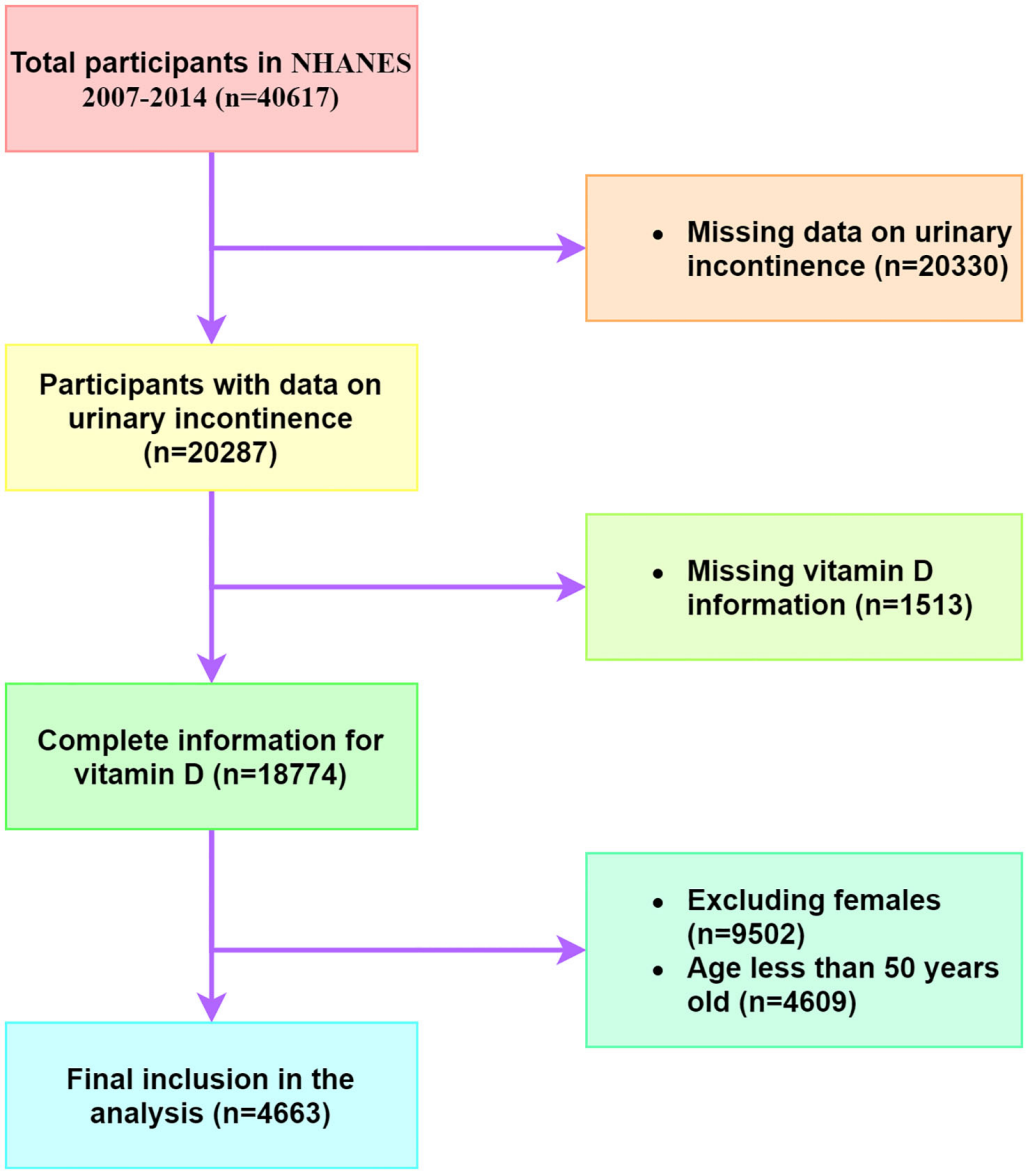
Flowchart for research.

The NHANES database is a publicly available repository of comprehensive health and nutrition data for the United States population. In this study, we extracted data from four NHANES cycles, spanning from 2007 to 2014, which contained consolidated serum 25(OH)D data. The study enrolled exclusively males aged 50 years and above, with exclusion criteria eliminating older men who did not provide urinary incontinence information or vitamin D data (**Figure 1**).

### Serum 25(OH)D levels

Standardized liquid chromatography-tandem mass spectrometry (LC-MS/MS) was utilized to measure serum 25(OH)D concentrations. It must be noted that total serum 25(OH)D levels below 50 nmol/L were classified as vitamin D deficient, while levels ranging from 50–75 nmol/L were considered vitamin D insufficient. On the other hand, levels of 75 nmol/L or higher were categorized as vitamin D sufficient, as per established guidelines (6).

### Definition of urinary incontinence

The assessment of urinary incontinence was conducted through the administration of a standardized questionnaire. In accordance with the criteria, any instance of urine leakage or loss of control during activities such as coughing, weight lifting, or exercise within the preceding 12-month period was classified as stress urinary incontinence (SUI). Urge urinary incontinence (UUI) was defined as involuntary urine leakage or loss of control as a result of the urge or pressure to urinate without the ability to reach a toilet in time. Concurrent manifestations of both SUI and UUI are categorized as mixed urinary incontinence (MUI).

### Covariates

In this study, hypertension is defined as having received a diagnosis of hypertension or having an average blood pressure of 140/90 mmHg or higher as indicated by a medical professional. Smoking status is categorized as either never, former, or current. Alcohol use is defined as having consumed at least 12 alcoholic beverages within one year. Depression status is evaluated using the PHQ-9, with a score of 10 or higher indicating depression. Additional covariates in the study include age categories (50–59, 60–69, and 70 or older), race, BMI (<25, 25–29.99, and 30 or higher), diabetes status (yes, no, or borderline), and family poverty ratio (<1.3, 1.3–3.49, and 3.5 or higher).

### Statistical analysis

Categorical variables were described using quantitative or proportional (%), while the mean ± standard error (SE) was used to express continuous variables. The analysis of the correlation between serum 25(OH)D and urinary incontinence was conducted utilizing weighted multivariate logistic regression models. Furthermore, in our research, we developed three models and determined their respective odds ratios (ORs). The first model was univariable and did not account for covariates, while model 1 adjusted for age, race, and BMI, and model 2 adjusted for age, race, BMI, alcohol use, diabetes, hypertension, family poverty rate, depression, and smoking. The level of statistical significance utilized in this study was determined to be a two-tailed p-value less than 0.05. The statistical analyses were conducted using software tools such as Stata and SPSS.

## Results

Our study included a total of 4663 older males who were selected for analysis. Out of these participants, 1263 were identified as vitamin D deficient, 1833 were classified as insufficient in vitamin D, and 1567 were found to have sufficient levels of vitamin D. A comprehensive overview of the study demographics can be found in **Table 1**. The prevalence of SUI, UUI, and MUI in the vitamin D deficient group was 8.3%, 26.8%, and 5.5%, respectively. The vitamin D insufficient group had a prevalence of 7.2%, 22.7%, and 4.3% for SUI, UUI, and MUI, respectively. Finally, the prevalence of SUI, UUI, and MUI in the vitamin D sufficient group had been recorded as 7.8%, 23.4%, and 4.4%, respectively.

**Table 1:** A comprehensive overview of the study demographics.

25(OH)D	< 50 nmol/l	50–75 nmol/l	≥ 75 nmol/l	P Value
**Participants (n)**	1263	1833	1567	
**Age, Mean ± SE**	62.68 ± 0.25	64.61 ± 0.22	66.87 ± 0.24	<0.001
**BMI, n (%)**				<0.001
<25	298(23.6)	356(19.4)	427(27.2)	
25-29.99	426(33.7)	761(41.5)	690(44.0)	
≥30	509(40.3)	699(38.1)	426(27.2)	
missing	30(2.4)	17(0.9)	24(1.5)	
**Race, n (%)**				<0.001
Non-Hispanic White	340(26.9)	919(50.1)	1064(67.9)	
Non-Hispanic Black	502(39.7)	301(16.4)	180(11.5)	
Other Hispanic	117(9.3)	199(10.9)	105(6.7)	
Other races	304(24.1)	414(22.6)	218(13.9)	
**Alcohol use, n (%)**				0.967
yes	1035(81.9)	1510(82.4)	1299(82.9)	
no	226(17.9)	321(17.5)	266(17.0)	
missing	2(0.2)	2(0.1)	2(0.1)	
**Hypertension, n (%)**				0.009
yes	812(64.3)	1108(60.4)	1004(64.1)	
no	434(34.4)	709(38.7)	556(35.5)	
missing	17(1.3)	16(0.9)	7(0.4)	
**Smoking, n (%)**				<0.001
never	452(35.8)	725(39.6)	579(36.9)	
former	449(35.6)	808(44.1)	758(48.4)	
current	360(28.5)	300(16.4)	230(14.7)	
missing	2(0.2)	0(0.0)	0(0.0)	
**Depression, n (%)**				0.004
yes	112(8.9)	101(5.5)	99(6.3)	
no	1127(89.2)	1705(93.0)	1442(92.0)	
missing	24(1.9)	27(1.5)	26(1.7)	
**Diabetes, n (%)**				0.027
yes	313(24.8)	368(20.1)	319(20.4)	
no	909(72.0)	1385(75.6)	1189(75.9)	
borderline	41(3.2)	78(4.3)	58(3.7)	
missing	0(0.0)	2(0.1)	1(0.1)	
**Family poverty ratio**				<0.001
<1.3	397(31.4)	449(24.5)	330(21.1)	
1.3-3.49	452(35.8)	654(35.7)	511(32.6)	
≥3.5	314(24.9)	584(31.9)	600(38.3)	
missing	100(7.9)	146(8.0)	126(8.0)	

**Figure 2:**
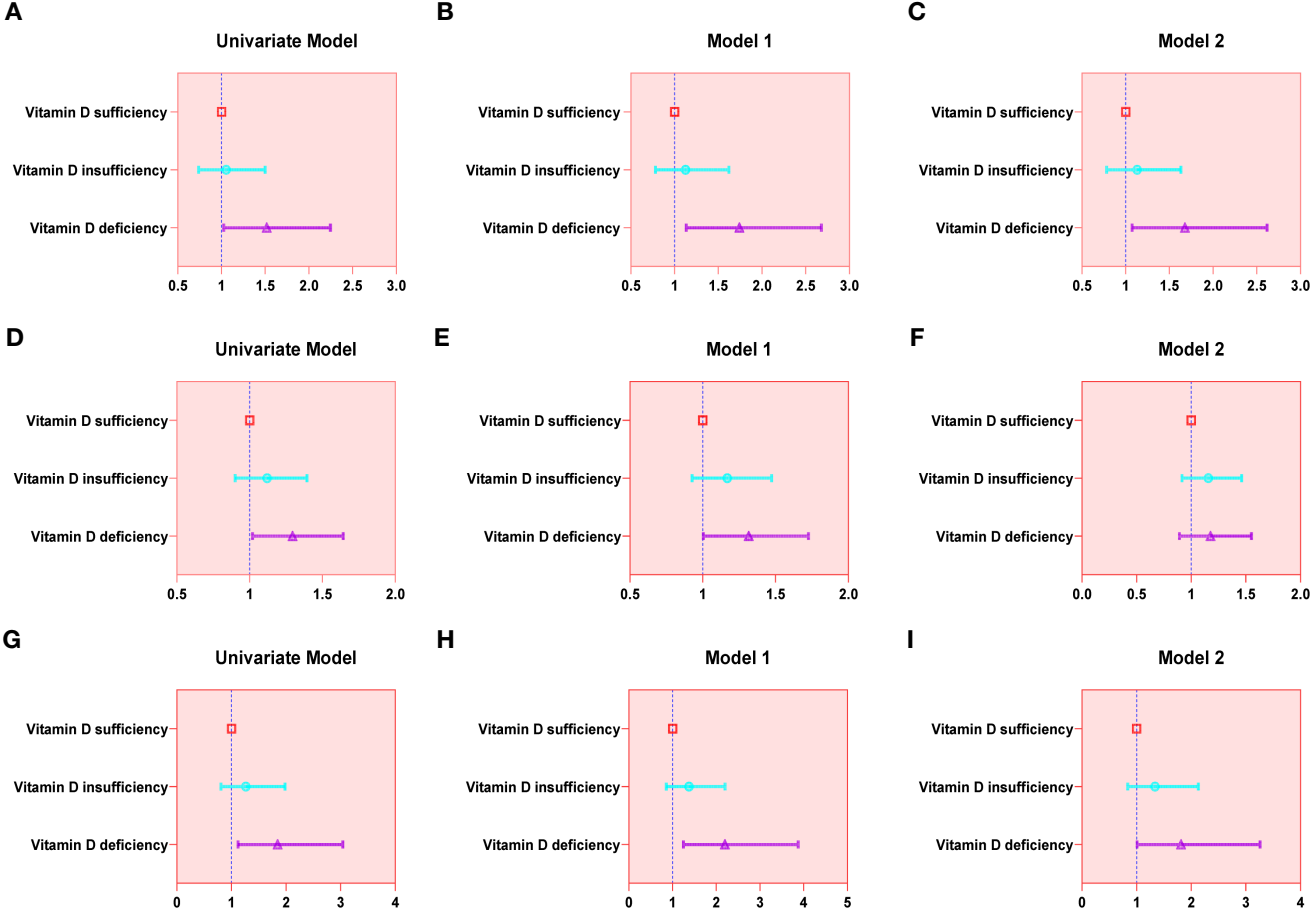
Forest plot for logistic regression analysis. **(A-C)**: SUI; **(D-F)**: UUI; **(G-I)**: MUI.

The findings of the univariable analysis revealed that the group with vitamin D deficiency exhibited augmented odds of all three urinary incontinence types in comparison to the vitamin D sufficient group. Upon making adjustments for age, race, and BMI, no significant changes in the results were observed. However, after accounting for all covariates, only SUI (OR = 1.677; 95% confidence interval (CI) = 1.074–2.618) and MUI (OR = 1.815; 95% confidence interval (CI) = 1.010–3.260) demonstrated statistical significance. A detailed account of the results can be referenced in **Table 2** and **Figure 2**.

**Table 2:** Results of weighted logistic regression analysis.

		SUI				UUI				MUI		
	Prevalences (%)	Univariable Model	Model 1	Model 2	Prevalences (%)	Univariable Model	Model 1	Model 2	Prevalences (%)	Univariable Model	Model 1	Model 2
25(OH)D												
<50 nmol/l	8.3	1.517 (1.025-2.246)	1.741 (1.132-2.679)	1.677 (1.074-2.618)	26.8	1.294 (1.020-1.642)	1.316 (1.004-1.725)	1.177 (0.893-1.550)	5.5	1.844 (1.119-3.039)	2.197 (1.246-3.873)	1.815 (1.010-3.260)
50-75 nmol/l	7.2	1.053 (0.739-1.500)	1.126 (0.782-1.621)	1.131 (0.783-1.632)	22.7	1.120 (0.900-1.393)	1.169 (0.927-1.473)	1.157 (0.916-1.462)	4.3	1.263 (0.806-1.981)	1.373 (0.857-2.201)	1.336 (0.838-2.130)
≥75 nmol/l	7.8	1.0 (Reference)	1.0 (Reference)	1.0 (Reference)	23.4	1.0 (Reference)	1.0 (Reference)	1.0 (Reference)	4.4	1.0 (Reference)	1.0 (Reference)	1.0 (Reference)

SUI, Stress urinary incontinence; UUI, Urge urinary incontinence; MUI, Mixed urinary incontinence.

Univariable Model: not adjusted; Model 1: adjusted for age, race, and BMI. Model 2: adjusted for age, race, BMI, alcohol use, diabetes, hypertension, family poverty rate, depression, and smoking.

## Discussion

The present study seeks to examine the correlation between serum 25(OH)D and urinary incontinence in elderly males. This study is novel in that it is the first national study to examine the association between serum 25(OH)D and various sub-types of urinary incontinence in older men. Our findings indicate that, in both the univariable model and model 1, vitamin D deficiency was linked with increased odds of urinary incontinence, including urge, stress, and mixed. However, model 3, which adjusted for all covariates, revealed that serum 25(OH)D was linked independently with stress and mixed urinary incontinence in older men. This discovery constitutes the most significant finding of our study.

Urinary incontinence and vitamin D deficiency were prevalent disorders in the elderly population. For females, there was substantial evidences supporting a strong association between vitamin D and urinary incontinence (13). A randomized controlled trial demonstrated that vitamin D supplementation improved urinary incontinence in premenopausal women (15). However, conflicting results have been reported that moderate doses of vitamin D supplements did not diminish urinary incontinence in older women, and the underlying causes of this discrepancy require further exploration (16). In contrast, research on the relationship between vitamin D and urinary incontinence in males was relatively limited. One previous study found a correlation between vitamin D deficiency and moderate to severe urinary incontinence in adult men, though this data may be subject to bias due to testing technology at the time (17). Moreover, they failed to investigate the relationship further with older men. Instead, the present study focused on older men and investigated the association between vitamin D deficiency and urinary incontinence in older men through a large national sample.

The precise mechanisms behind the correlation between vitamin D and urinary incontinence in elderly men have yet to be fully elucidated and require further investigation. Nonetheless, multiple mechanisms may potentially be at play. Initially, it was recognized that the processes that govern urination were intricate and predominantly governed by neural, muscular, and other influences. Studies on neuromodulation evidenced that vitamin D plays a protective role by elevating antioxidant levels in neurons (18). Furthermore, vitamin D has been found to regulate the expression of several neurotransmitters, including acetylcholine and dopamine (18, 19). Also, as demonstrated in *in vitro* experiments, 1,25-dihydroxyvitamin D3 (1,25(OH)2D3) was effective in mitigating neuroinflammation through the inhibition of MAPK pathways, as well as promoting neural stem cell proliferation and enhancing their differentiation into neurons and oligodendrocytes (20, 21).

Secondly, the presence of vitamin D receptors throughout various tissues and organs, such as muscle, bladder, prostate, and urethra, has been well established (22). Recent studies have demonstrated that vitamin D has an impact on muscle strength and function by influencing various cellular processes such as cell proliferation, differentiation, protein synthesis, and myotube size (23). Experiments *in vitro* have displayed that the signaling of vitamin D and the vitamin D receptor effectively abrogated skeletal muscle atrophy through its inhibitory effect on the renin-angiotensin system (24). Furthermore, deficient levels of vitamin D may give rise to an array of consequences. Such consequences may encompass the deregulation of calcium metabolism, which can lead to anomalous contraction of the detrusor muscle. Abnormalities of the detrusor muscle are usually one of the most significant factors causing urinary incontinence, and surgery can improve the associated symptoms to a certain extent (25). And some studies have reported that a postoperative combination of Ospemifene can significantly improve patients’ symptoms without increasing postoperative adverse events (26). Additionally, recent findings suggest that low levels of vitamin D may facilitate increased inflammatory cytokine activity, provoking inflammation in the bladder wall and thereby impairing urinary function (27).

In addition to its direct impact, vitamin D may also have an indirect influence on urinary incontinence through various means. Benign prostatic hyperplasia (BPH) was a frequent contributor to bladder outlet obstruction and a significant factor in urinary incontinence experienced by men in their senior years (28). Several research studies have pointed out a possible correlation between vitamin D deficiency and BPH. Vitamin D supplementation, on the other hand, has been suggested as a potential option to alleviate BPH symptoms. To elaborate, VDR agonists like elocalcitol could potentially enhance bladder contractility and obstruct prostate enlargement, thereby alleviating BPH symptoms (29). Furthermore, low serum vitamin D levels have been attributed to an increased risk of falls in old age, which could possibly increase the risk of urinary incontinence to some extent. Other than that, evidence-based medicine pointed out that individuals suffering from depression encountered more severe urine incontinence symptoms. According to one study, vitamin D supplementation may alleviate depression (30, 31). Beyond that, there may be other mechanisms, so more research was required to explore them.

Finally, this research presents various advantages. At first, it represents the first nationwide research assessing the relationship of serum 25(OH)D with distinct sub-types of urine incontinence in older males. In the second place, we incorporated data from four cycles, yielding a huge sample size. And third, serum vitamin D in the present investigation was detected *via* the LC-MS/MS method, which provided higher accuracy compared to previous assay techniques. Lastly, in the multi-factorial logistic regression analysis, we controlled for plenty of variables. However, several flaws remained. This was an observational study, and even though we controlled for numerous confounders, we couldn’t rule out the influence of any unmeasured confounding factors. Additionally, attrition bias may exist. Hence, further studies are still required to confirm the causal relationship between them and the underlying mechanisms.

## Conclusion

Decreased serum 25(OH)D levels were connected with stress urinary incontinence and mixed urinary incontinence in elderly men.

## Data availability statement

The original contributions presented in the study are included in the article/Supplementary Material. Further inquiries can be directed to the corresponding author.

## Author contributions

LL and MX conceptualized the design of this work. XH and XC were engaged in collecting and organizing the data. The data processing and image creation were executed by MX and HZ. The initial version of the paper was accomplished by MX and LL. XL was in control of reviewing and proofreading the manuscript. All authors contributed to the article and approved the submitted version.
